# Prediction of intention to continue sport in athlete students: A self-determination theory approach

**DOI:** 10.1371/journal.pone.0171673

**Published:** 2017-02-08

**Authors:** Mohammad Keshtidar, Behzad Behzadnia

**Affiliations:** 1 Faculty of Physical Education and Sport Science, University of Birjand, Birjand, Iran; 2 Department of Motor Behaviour, University of Tabriz, Tabriz, Iran; University of Rome, ITALY

## Abstract

Grounded on the self-determination theory (Deci & Ryan, 1985, 2000) and achievement goals theory (Ames, 1992; Nicholls, 1989), this study via structural equation modelling, predicted intention to continue in sport from goal orientations and motivations among athlete students. 268 athlete students (*M*_age_ = 21.9), in Iranian universities completed a multi-section questionnaire tapping the targeted variables. Structural equation modelling (SEM) offered an overall support for the proposed model. The results showed that there are positive relationships between intention to continue in sport and both orientations as well as both motivations. A task-involving orientation emerged as a positive predictor of the autonomous motivation, while an ego-involving orientation was a positive predictor controlled motivation as well as autonomous motivation. The results also support positive paths between autonomous motivation and future intention to participate in sport. Autonomous motivation also was a positive mediator in relationship between task orientation and the intentions. As a conclusion, the implications of the task-involving orientation are discussabled in the light of its importance for the quality and potential maintenance of sport involvement among athlete students.

## Introduction

The importance and benefits of exercise in health and well-being cannot be denied [1–3]. However, research evidence indicated that exercise and engaging in physical activities declines with age, and national organizations such as US Department of Health and Human Services and University Sport Organization in Iran (2013) have recommended that school and college physical education programs should play a crucial role in engaging and adhering the physical activities among students [4].

Many motivational theories proposed models and mechanisms by different constructs affect physical activities adherence. However, self-determination theory (SDT) [5, 6] and achievement goal theory (AGT) [7, 8] received empirical supports, because of their tents world-while accepted and they could describe the effects of social-contexts determinations on individual functions, the desire to participate in physical activities. More specific, SDT provides a more complete picture of human motivation and personality than do various other theories that emphasize just one necessary dimension of social-contexts [5, 6, 9]. The purpose of current study was to further understanding college-students’ intentions to continue sport based on the type of orientations and motivational regulations. We first detail the theoretical background of both AGT and SDT and their central characteristics, and then refer to more recent findings about them and participation in physical activities.

### Achievement goal theory

AGT was developed to consider the effects of perceptions of success versus failure on motivation, which aims to understand individual’s view of competence and the role of environment or motivational orientations [7, 8]. Based on AGT, a person may hold two distinct dispositional motivational orientations. Individual with a self-referenced or task orientation tend to view success and failure in terms of self-mastery goals and learning in which describing one’s competence. Instead, people with an other-referenced or ego orientation tend to compare their success or failure with others, that is based on other-referenced goals and demonstrating superior ability. Moreover, task-involved people are more likely to choose to engage and take interesting in the activity, conversely, ego-involved people tend to focused on comparisons, so that they lose interest in the activities and reduced motivation to engage with it in future [10, 11]. Therefore, these orientations affect individual interpretation of competence across variety domains, such as physical activity [12, 13].

### Self-determination theory

SDT is a macro theory in human motivation and personality that describe the effects of social-contexts determinations on human motivation and functions. SDT propose a continuum of motivational regulations from intrinsic motivation to extrinsic motivation and amotivation. The two general categories of motivation include autonomous motivation (intrinsic motivation, integrated and identified regulations) and controlled motivation (interjected and external regulations). Each of these motivations, considering the rate of autonomy, is placed on a continuum limit ranged from low autonomous (external motivation) to high autonomous (intrinsic motivation). Autonomous motivation includes the person's involvement in an activity based on the willingness and decision of that person, retention of certain behavioural results that are important for the person such as promotion of physical fitness and health and enhancement of sport performance; and also there are factors with most autonomous behaviour in which the participation of the individual is for sake of the sense of enjoyment or pleasure. In this type of motivation, participation in a particular activity is because of interpersonal values and commitment of the individual. In contrast, the controlled motivation shows the participation of the individual in an activity to achieve the desired results such as a reward or avoiding punishment, as well as stress, sense of guilt or self-enhancement. In this type of motivation, doing of the task is carried out with a feeling of pressure and the environmental forces [5, 9, 14].

From the perspective of SDT, motivation to exercise and sport is a complex phenomenon, because people have multiple motivations to participate in sport and exercise activities [5, 6, 9]. For instance, athletes can be motivated by rewards, assessments, pressure from parents or educators or attitudes that others have towards them [15]. They also can progress in sport by interest, curiosity or the desire for domination. Additionally, SDT provides a comprehensive framework for understanding the intrinsic and extrinsic motivators and their impact on stability in sport and their different effects on benefits resulted from sport [16, 17]. Based on SDT, motivational structures between men and women are constant [5, 6], however, because of the study population (i.e., athlete students), we measured firstly gender differences to whether considering gender as a moderate variable in the analyses data.

Previous research indicated that intrinsic motivation is related with positive outcomes in education and sport domains [18, 19]. Sarrazin Vallerand, Guillet, Pelletier and Cury (2002), through structural equation modelling, showed that low levels of autonomous motivation were related to dropping out of sport, so that this level of behavioral control was associated with dropping out of an activity. In contrast, the high levels of autonomous motivation were associated with female athletes’ behavioral intentions [20]. Alvarez, Balaguer, Castillo and Duda (2012) also showed that intrinsic motivation was positively associated with the players' intention to continue the sport in the future [21]. In this study, we aim to examined motivational regulations at university sports. We expected that autonomous forms of motivation are related to students’ behaviours to continue sport in future.

### Integrating the theories

The framework of these two theories emphasizes on the principle of social-contextual determinations associated with changeability of people motivation level, and the requirements of such motivational differences are due to the quality of behaviors and individual's intentions to participate in specific domains. General convergence of both SDT and AGT suggest that the use of mastery goals or “performance-based rewards” and social comparisons as motivational strategists produce many different concealed costs. Both theorists suggest that environments that support people’s autonomy and intrinsic desires provide the basis for positive outcomes such as well-being and enhanced achievement [6].

Research has illustrated that mastery goals are most strongly related to positive outcomes such as perceived competence [10, 22], autonomous motivation [12, 23], enjoyment [24] and behavioural persistence [25]. Research also in the area of physical activities showed that mastery orientated goals positively related to autonomous motivation, whereas negatively related to controlled regulations [26]. Using these theories could provide useful helps for researchers as well as teacher and sport instructors in determining these motivational factors at university. Moreover, several research has identified similarities between these theories, in which task orientation can enhance autonomous motivation, conversely, ego orientation associated with controlled motivation [12, 27].

### The present study

In the present study, we aim to investigated an integrated model incorporating AGT and SDT to examine influences on college students’ intentions to continue sport in future, using structural equation modelling. As outline previously, intentions to continue sport in future can be considered as an outcome of both sets of motivational constructs. Previous research has indicated positive relationship between participation in sport and goal orientation, but it is still unclear whether goal orientations is related to college students’ intention to continue sport in future. Previous researches had focused more on the club athletes and physical education [12, 21, 22, 27]. However, importantly, the present study focus on participation of sport activates outside of the university or next semesters/months rather than just focusing on physical education.

The proposed model of this study will contribute to present knowledge by establishing how goal orientations is translated into sport intentions at university sports. Moreover, the results of this study can determine that which type of motivation and goal orientations can influence on the intention to continue sport and more persistence in sport among athlete students. In terms of implications for university sports, the model will provide evidence that goal orientations are those that are more likely to foster autonomous motivation and behavioural intentions in athlete students. The findings of this study also can develop the theoretical literature existing in this field and provide evidence for relationship of the AGT and SDT at university sports.

Importantly, it is crucial to consider motivational regulations as mediators between goal orientations and students’ intention, as SDT postulated that people’s motivation can determine their intentions and behaviours [5, 6]. Therefore, in this study we constructed structural equation model investigating the casual relationship between athlete students’ orientations, motivational regulations and intention to continue sport in future, respectively. More specifically, we investigated whether the effects of goal orientations and future sport behaviour are mediated through motivational regulations.

## Method

269 athlete students (57 female students and 212 male students) from two universities in Iran were randomly selected. The mean age of participants was 21.93 years old (SD = 3.17). Excluding criteria pertained to have regular sport trainings three times per week for at least six months. The average duration of sport activities was also 0.86 years old (SD = 2.15). Four data were not included in the study due to non-filled scales. Prior to the start of gathering data for the study, written informed consent and assent was obtained. Also, ethics guidelines for behavioural research approval was granted by the Birjand University’s Ethical Board.

### Measurements

#### 1. Autonomous and controlled motivation

To measure autonomous and controlled motivation, the behavioral regulation in sports questionnaire (BRSQ-6) [28], which include six factors (amotivation, external regulations, introjected regulations, identified regulations, integrated regulations and intrinsic motivation) with 24 items, was used. In this questionnaire, based on the purposes of the study, the components of external regulations and interjected regulations as the indices of controlled motivation (e.g., “because I feel pressure from other people to play” and “because I would feel guilty if I quit”), and the components of integrated regulations and intrinsic motivation as the indices of autonomous motivation (e.g., “because it’s a part of who I am” and “because I enjoy it”) were measured [29, 30], which is a 16-items. The stem of all items begins with the statement "I participate in this sport, because…”. Scoring was done in 7-point Likert type scale (7 = completely right, and 1 = completely wrong). Validity and reliability of the questionnaire has been confirmed among athlete students in Iran by Ahmadi, Behzadnia and Amani (2016) [23]. Cronbach's alpha coefficient for autonomous and controlled motivation was calculated equal to 0.70 and 0.75, respectively.

#### 2. Intention to continue sport in future

Intention to continue sport in the following months/terms was assessed using three items based on the research by Chatzisarantis and colleagues (1997) [31]. The participants responded to three questions as following: "I have decided to continue this sport activity in the next month’s/semesters"; "I am going to do this sport activity in the next month’s/semesters"; "I have plan for sport activity in the next month’s/semesters". Scoring was done in 7-point Likert type scale (7 = very high, and 1 = very low). Previous studies have indicated an acceptable internal reliability in sport [27]. This questionnaire also had a good validity in Iranian samples [32]. Cronbach's alpha coefficient for the questionnaire was obtained equal to 0.82.

#### 3. Task and ego orientation in sport questionnaire

Task and ego orientations in sport questionnaire [33] were used in this study. The questionnaire is a 13-items which 7 items are related to task orientation (e.g., “I learn something that is fun to do”), and 6 items related to ego orientation (e.g., “The others can’t do as well as me”), with 5-point Likert type scoring scale (1 = completely disagree, 5 = completely agree). The stem for each item is: I feel most successful in sport when …”. Reliability and validity of the questionnaire was measured among Iranian sample by Shafi'zadeh [34]. In current study, Cronbach's alpha for the ego orientation and task orientation was obtained equal to 0.69 and 0.75, respectively.

### Data analysis

Independent t-test firstly was measured to whether consider gender as a moderate variable in the relationship between goal orientation with motivational regulations and behavioural intentions. The hypothesized effects of goal orientations and motivational regulations on behavioural intentions was tested using structural equation modelling with Amos software version 20. The chi square test (*χ*^2^), comparative fit index (CFI), root mean square error of approximation (RMSEA), tucker lewis index (TLI) and standardized root mean square residual (SRMR) were used to evaluate the adequacy of the model.

Measuring the effects of the mediators' roles was based on Preacher and Hayes model [35]. This model is based on a normal theoretical approach and bootstrapping approach for obtaining reliable intervals between variables [36, 37] and this approach supports the traditional methods of Baron and Kenny (1986) in studying the effects of the role of mediating variables [38]. Statistical difference was accepted at *p*<0.05.

## Results

Table 1, shows descriptive statistics (i.e., mean, standard deviation) of variables. In casual modelling, distribution of variables should be normal [38]. Kline (2005) proposed that the absolute value of skewness and kurtosis of variables should not be higher than 3 and 10 respectively [38] The absolute values of skewness and kurtosis of all variables were lower than values mentioned by Kline (range from -0.18 to 5.57). So, this default for casual modelling, that is, univariate normality is restored in the research. Independent t-test results showed that gender does not have a significant effect on the tested variables (see S1 Data, that is a SPSS file).

**Table 1:** Descriptive statistics and the correlation matrix between variables.

	Variable	1	2	3	4	5
1	Task orientation	1				
2	Ego orientation	0.34**	1			
3	Autonomous motivation	0.42**	0.27**	1		
4	Controlled motivation	0.03	0.30**	0.15*	1	
5	Intention to continue sport	0.21**	0.24**	0.37**	0.12*	1
	*Mean*	4.03	3.38	4.77	2.93	4.73
	*SD*	0.64	0.74	0.58	1.13	0.91

Note:

* = p<0.05,

** = p<0.01.

In Table 1 also, the results of the correlation matrix between variables are shown. According to the results, there is a positive significant relationship between task orientation with autonomous motivation and intention to continue sport. Also there is a positive significant correlation between ego orientation with autonomous and controlled motivation, and also with intention to continue sport. The autonomous motivation and controlled motivation also positively related to intention to continue sport.

**Fig 1:**
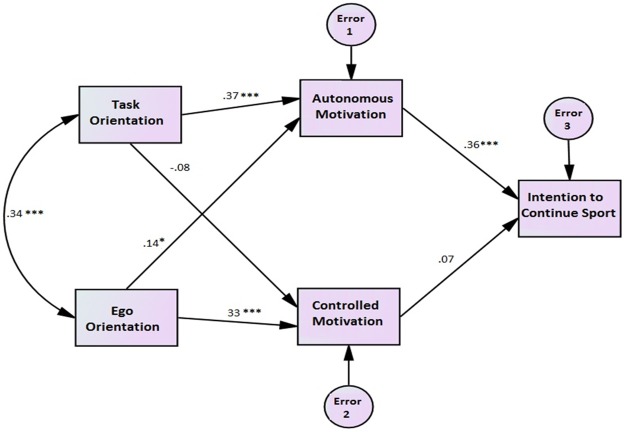
Research model in path coefficient mode.

The structural model demonstrated a reasonable fit to the data, *χ*2 (3) = 8.7, p < .03; CFI = .96; RMSEA = .08; RMSEAR 95% CI = .02 to .15; TLI = .88; SRMR = .04. Significant path coefficients (standardized estimated) are presented in Fig 1.

As shown in the Fig 1, the model is generally confirmed. Task orientation significantly and positively predicts autonomous motivation and this type of motivation significantly predicts the intention to continue sport in future. Ego orientation significantly predicts both controlled motivation. and autonomous motivation (*p*<0.05).

The results of mediation role showed that the overall effect of task orientation on the intention to continue sport was significant (t = 3.5; p<0.001), while there was no direct and significant impact between these two variables, and autonomous motivation was a positive mediator in the relationship between task orientation and intention to continue sport (*p*<0.001; *F* = 15.07; R-sq = 0.15). The results also showed that ego orientation only had a direct impact on the intention to continue sport and the roles of mediators were not significant.

## Discussion

The aim of present study was to predict the intention to continue sport in athlete students based on the type of goal orientations and motivation indicators. This research was carried out based on the approaches of SDT and AGT. Firstly, the results showed that there was a significant correlation between the two theories, as Deci and Ryan (2000) had proposed on [6].

Results in line with the SDT [5, 6, 9] and AGT [7, 8] in athlete students showed that there was a positive relationship between task orientation with autonomous motivation and intention to continue to sport in the following semester/months. This result is also consistent with the results of study of Alvarez et al. (2012) that emphasized on the positive relationship between task orientation with intrinsic motivation and intention to continue sports [21]. Findings also showed that the orientation based on the intention and needs of athlete students had a relationship with autonomous motivation indicators, that is, participation in sport is based on interest and pleasure and these desires had a positive correlation with intention to continue sport it in the following months/semesters. Interestingly, the results indicated that ego orientation had a relationship with both autonomous and controlled motivation and intention to continue sport. This association inversely showed by Alvarez et al. (2012) that ego orientation was negatively associated with intrinsic motivation [21]. This association had not been indicated in this form by previous research. This relationship could be due to the positive relationship between the two types of orientation with each other, while based on the AGT [7, 8] these two types of orientation must have negative or inverse relationship with each other and this association has been clearly defined in the previous research [21]. In contrast to the AGT and previous research, and align with the current findings, Hein and Hagger (2007) reported a strong correlation between ego and task orientations among young school children [12].

Based on the current results, both goal orientations are directly compatible with autonomous forms of motivation, but somewhat different in correlation amounts, because the context of university sports have shown to enhance students’ autonomous motivation. It seems both goal orientations promote more engaging and exercise adherence as well as are attributable to the context of enhancing autonomous motivation. Moreover, ego orientation also is directly compatible with controlled forms of motivation and it seems an ego orientation also promotes students’ intentions to engaging in sport activities in future.

While both autonomous and controlled motivation positively associated with the intention to continue sport in future, autonomous motivation related more with behavrional intention in compared with controlled motivation. Interestingly, both autonomous and controlled motivation involved, but somewhat different, the intentions to continue sport in future among college students. Unlike with previous research that highlighted the controlled forms of motivation negatively related to behavioural intentions among secondary school students [39] and physical education [40], the current results illustrated that the controlled motivation positively related to intentions. Therefore, our hypothesis partially supported, which both types of behavioural regulations influence students’ intention, with the strongest correlation for autonomous motivation.

In keeping with finding from previous research, task orientation positively predicted autonomous motivation, and in turn the autonomous motivation was a positive predictor of intention to continue sport among students. Importantly, autonomous motivation is responsible for the translation of task orientation at university sports into sport involvement and intentions and supports the multi-theory approach adopted by the current study. Thus, the current result confirms and extends the assumptions relating to both SDT [5, 6, 9] and AGT [7, 8] at university sports. This finding also supports Deci and Ryan's (2000) views in the relationship between the SDT and AGT, at university sports [6].

Additionally, ego orientation positively predicts controlled motivation that this result refers to the relationship between comparing individuals' activities based on external motivation and the use of force by the others to participate in a sport activity. However, it might be due to the samples of the present research or students’ perspectives at university sports, which needs further research in this field.

Finally, the last study’ hypothesis also supported that autonomous motivation positively mediates the relationship between task orientation and the intention to continue sport among college students, and this support the finding of researchers that have used the integrating of SDT and AGT to explain the intentions [6, 10, 12, 31]. Moreover, in contrast, the controlled motivation did not have an effective role in this prediction. Thus, the significant total effect of autonomous motivation at university sports on sport intentions can explain through the task orientation and the SDT and AGT. More advanced models that include constructs which not yet exploited by the behavioural regulations may provide an analog to explanation of students’ behavioural intentions.

## Conclusion

The present study provides support for a model of the goal orientations effects on motivational regulations and behavioural intentions at university sports. These motivational regulations explain behavioural intentions and contributes to the understanding of the processes of considering needs-related motives expressing motivated actions. These results suggest that an important step in facilitating students’ autonomous motivation to participate and intention to continue sport activities may begin in university. Teachers therefore may facilitate adopting an appropriate motivational discourse by providing appropriate behaviours and feedbacks to enhance autonomous motivation for physical activity [7, 41].

The external or controlled forms of motivation may explain, perhaps, because students are not able to choose activities that gradually (and firmly) feelings of competence and interest during sport activities, then students may focus on deriving competence and enjoyment form participating in alternative sport activities that she or he has chosen. Moreover, previous empirical research could support the current finding that controlled forms of motivation in schools was also positively associated with the intentions to continue physical activities in future [31]

## Supporting information

S1 Data That is a SPSS file. (SAV)
